# [^64^Cu]Cu-DOTATATE PET metrics in the investigation of atherosclerotic inflammation in humans

**DOI:** 10.1007/s12350-022-03084-4

**Published:** 2022-08-31

**Authors:** Jacob K. Jensen, Johanne S. Madsen, Malte E. K. Jensen, Andreas Kjaer, Rasmus S. Ripa

**Affiliations:** 1 Department of Clinical Physiology and Nuclear Medicine, Copenhagen University Hospital – Rigshospitalet, Blegdamsvej 9, 2100 Copenhagen, Denmark; 2 Cluster for Molecular Imaging, Department of Biomedical Sciences, University of Copenhagen, Copenhagen, Denmark

**Keywords:** inflammation, atherosclerosis, PET, molecular imaging, ASCVD

## Abstract

**Purpose:**

The aim of this study was to assess and compare the arterial uptake of the inflammatory macrophage targeting PET tracer [^64^Cu]Cu-DOTATATE in patients with no or known cardiovascular disease (CVD) to investigate potential differences in uptake.

**Methods:**

Seventy-nine patients who had undergone [^64^Cu]Cu-DOTATATE PET/CT imaging for neuroendocrine neoplasm disease were retrospectively allocated to three groups: controls with no known CVD risk factors (n = 22), patients with CVD risk factors (n = 24), or patients with known ischemic CVD (n = 33). Both maximum, mean of max and most-diseased segment (mds) standardized uptake value (SUV) and target-to-background ratio (TBR) uptake metrics were measured and reported for the carotid arteries and the aorta. To assess reproducibility between different reviewers, Bland–Altman plots were made.

**Results:**

For the carotid arteries, SUV_max_ (*P* = .03), SUV_mds_ (0.05), TBR_max_ (*P* < .01), TBR_mds_ (*P* < .01), and mean-of-max TBR (*P* = .01) were overall shown to provide a group-wise difference in uptake. When measuring uptake values in the aorta, a group-wise difference was only observed with TBR_mds_ (*P* = .04). Overall, reproducibility of the reported uptake metrics was excellent for SUVs and good to excellent for TBRs for both the carotid arteries and the aorta.

**Conclusion:**

Using [^64^Cu]Cu-DOTATATE PET imaging as a marker of atherosclerotic inflammation, we were able to demonstrate differences in some of the most frequently reported uptake metrics in patients with different degrees of CVD. Measurements of the carotid artery as either maximum uptake values or most-diseased segment analysis showed the best ability to discriminate between the groups.

**Supplementary Information:**

The online version contains supplementary material available at 10.1007/s12350-022-03084-4.

## Introduction

Despite advances in medical and interventional therapies in recent decades, morbidity and mortality due to ischemic disease like myocardial infarction and stroke remain high.^[1,2]^

Today, clinically applied non-invasive measures of the atherosclerotic burden like computed tomography (CT) calcium scoring offers improved risk stratification and selection of treatment strategy.^[3]^ Positron emission tomography (PET) imaging has the distinct advantage of being able to selectively image and quantify, depending on tracer choice, ongoing physiological processes, e.g., inflammation, hypoxia, or calcification.^[4]^ In the context of inflammation and atherosclerosis, macrophages are of special interest because of their essential role in the development of atherosclerosis.^[5]^ The majority of previous research in this context has been done using [^18^F]-fluoro-deoxyglucose ([^18^F]FDG), a marker for tissue metabolism and glycolysis, measuring ongoing inflammation as increase in arterial wall glucose metabolism.^[6–8]^ This has offered the opportunity to noninvasively quantify the effect of interventions on vascular inflammation.^[9,10]^

Other, more specific radiotracers aimed at investigating macrophage activity and inflammation are continually being developed and evaluated. Inflammatory M1 macrophages have been shown to express the G-protein-coupled receptor somatostatin receptor subtype-2 (SST_2_), which is the target of [1,4,7,10-tetraazacyclododecane-*N*,*N*′, *N*″,*N*′′′-tetraacetic acid]-D-Phe1, Tyr3-octreotate (DOTATATE), a SST_2_ ligand. DOTATATE labeled with either copper-64 (^64^Cu) or gallium-68 (^68^Ga) is used clinically in the diagnostic evaluation of neuroendocrine neoplastic disease (NEN), but is also being increasingly adopted in research as a marker of atherosclerotic inflammation. Both preclinical and clinical trials have demonstrated that PET imaging of vascular inflammation using radioactive-labeled DOTATATE is feasible.^[11–15]^ ^64^Cu labeling has distinct advantages that are of particular importance when imaging atherosclerosis, this being a shorter positron range (^64^Cu: ~ 1 mm vs ^68^Ga: ~ 4 mm), allowing for improved PET image resolution, as well as longer half-life (12.7 hours), which offers the possibility for image acquisition at later time points.^[16–18]^ However, information about (1) the most appropriate method to quantify [^64^Cu]Cu-DOTATATE uptake in atherosclerosis and (2) optimal cut-point values to distinguish between patients with no known cardiovascular disease (CVD) and those with known disease. This information is vital when designing clinical studies using [^64^Cu]Cu-DOTATATE as a tool to quantify arterial inflammation.

The purpose of this study was to retrospectively assess arterial uptake of [^64^Cu]Cu-DOTATATE in the aorta and carotid arteries and compare commonly used PET metrics. To do so, a cohort of patients was divided into 3 distinct groups, based on differences in cardiovascular risk profile at time of imaging: (1) patients with no known cardiovascular risk factors, (2) patients with risk factors for CVD, and (3) patients with a history of a previous ischemic event (myocardial infarction, transient ischemic attack, or ischemic stroke). We also aimed to assess the reproducibility of the [^64^Cu]Cu-DOTATATE PET uptake metrics most commonly used in atherosclerosis research.

## Materials and methods

### Patient cohort

**Figure 1:**
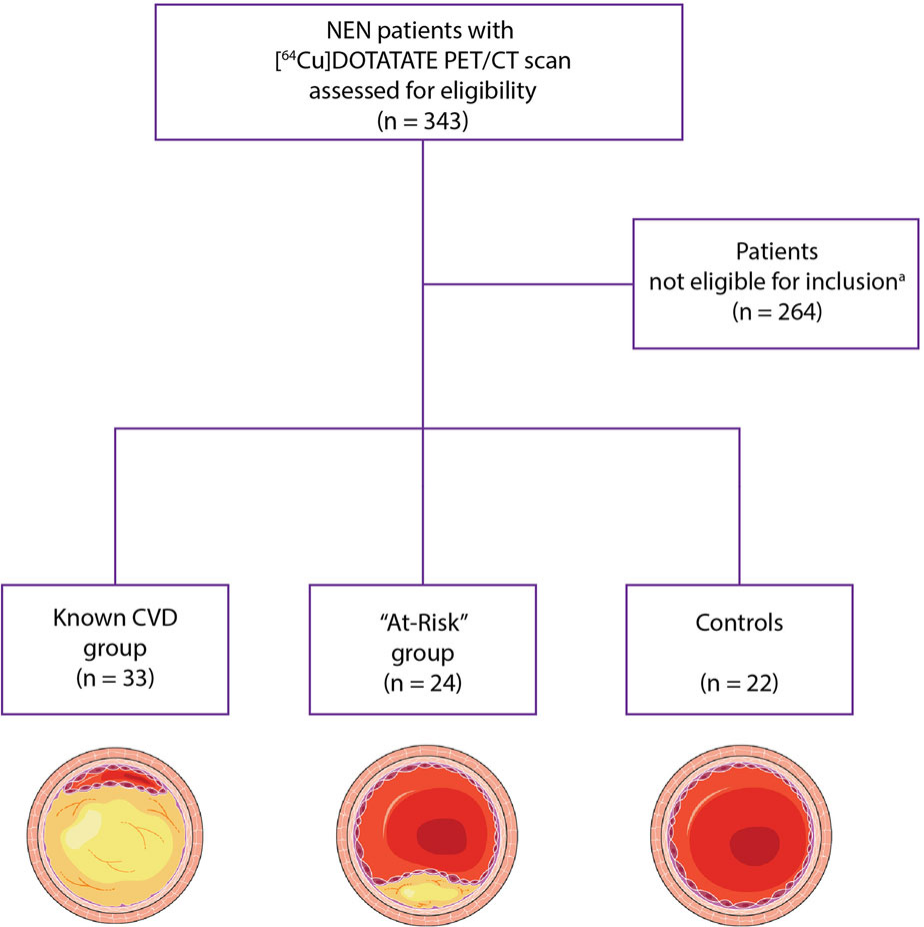
“Flowchart of inclusion and allocation into the 3 groups.” ^a^Patients that were not eligible for inclusion in any of the groups due to the following: Age < 50 years, PET/CT scan not available for analysis, PET/CT scan reconstructed using a different algorithm than specified, demographic variables of interest not available in the electronic medical record, use of one or more of prespecified immune-regulatory drugs, and PET/CT scan not performed within the timeframe of approval. *CVD*, cardiovascular disease; *CT*, computed tomography; *NEN*, neuroendocrine neoplasia; *PET*, positon emission tomography

All individuals who had undergone routine clinical [^64^Cu]Cu-DOTATATE PET/CT, at Department of Clinical Physiology, Nuclear Medicine and PET, University Hospital Rigshospitalet due to suspected or known NEN in the time period from November 2014 until February 2021 were retrospectively identified and assessed for eligibility for inclusion. The criteria for inclusion were as follows: Referral for suspected NEN diagnosis or staging of NEN, age > 50 years at time of PET/CT scan, [^64^Cu]Cu-DOTATATE whole-body PET/CT scan performed within the timeframe of the study approval and available for analysis, and no treatment with immune-modifying medication (glucocorticoids or conventional and biologic disease-modifying antirheumatic drugs) one year prior to the [^64^Cu]Cu-DOTATATE PET/CT scan (Figure 1).

In Denmark, all individuals are given a permanent unique personal identification number from the National Central Population Register at birth. All medical procedures, treatments, hospital admissions, and diagnoses are registered in relation to the patient’s unique personal identification number in an electronic medical record (EMR) system. Hospital admissions are registered with a primary, and if applicable, secondary diagnosis according to the 10th revision of the International Classification of Diseases (ICD-10).

Data regarding prior diagnoses, medical treatments, procedures, and diagnostic testing were collected from electronic medical records using the unique personal identification number.

Comorbidities occurring prior to the PET/CT scan were identified using primary and secondary diagnoses registered in the EMR system. Comorbidities registered were diabetes mellitus, hypertension, and hyperlipidemia. The use of antihypertensive or lipid-lowering drugs at the time of the PET/CT scan was used as a proxy for a diagnosis of hypertension and hyperlipidemia if no primary or secondary diagnosis was registered in the EMR. Other demographic variables of interest collected were age, body mass index (BMI), sex, and smoking history.

Included patients were allocated to one of three groups: (1) a group of control patients comprised patients with no registered cardiovascular risk factors (diabetes mellitus type 2, hypertension, or hyperlipidemia) or known treatment thereof. (2) An “At-Risk” group of patients with one or more of the previously mentioned risk factors, but no confirmed previous ischemic event (myocardial infarction requiring either percutaneous coronary intervention or coronary artery bypass graft surgery, transient ischemic attack, or ischemic stroke). Patients in the “At-Risk” group were selected for availability of laboratory recorded values of plasma total cholesterol, high-density lipoprotein cholesterol, and systolic blood pressure measurements performed in a period of up to 6 months prior to the PET/CT scan, to allow for calculation of the 10-year Framingham risk score for cardiovascular disease.^[19]^ (3) A group consisting of patients who had previously, prior to the [^64^Cu]Cu-DOTATATE PET/CT scan, experienced a minimum of one confirmed ischemic event.

If more than one [^64^Cu]Cu-DOTATATE PET/CT was available, the earliest was used for assessment.

Approval for this study and the disclosure of personal medical record data without informed consent were given by the Danish Patient Safety Authority (approval no: 31-1521-152) and did not require individual consent from the patients included.

### Image acquisition and analysis

All included patients had whole-body PET/CT scans performed on a Siemens Biograph mCT 64 True Point hybrid PET/CT medical imaging system available for analysis. PET images were acquired 60-minute post-injection of 200 MBq [^64^Cu]Cu-DOTATATE. Images were reconstructed using the same standard algorithm used clinically (Point spread function, TrueX; Siemens Medical Solutions) using 3 iterations, 21 subsets, and a 2-mm Gaussian filter for smoothing (full width at half maximum). PET images were reconstructed using a low-dose non-contrast-enhanced CT-based attenuation correction. Diagnostic quality venous phase CT scans (slice thickness 2 mm) using iodine intravenous contrast was performed in succession with the PET scan and used for region of interest (ROI) tracing of the aorta and carotid arteries.

**Figure 2:**
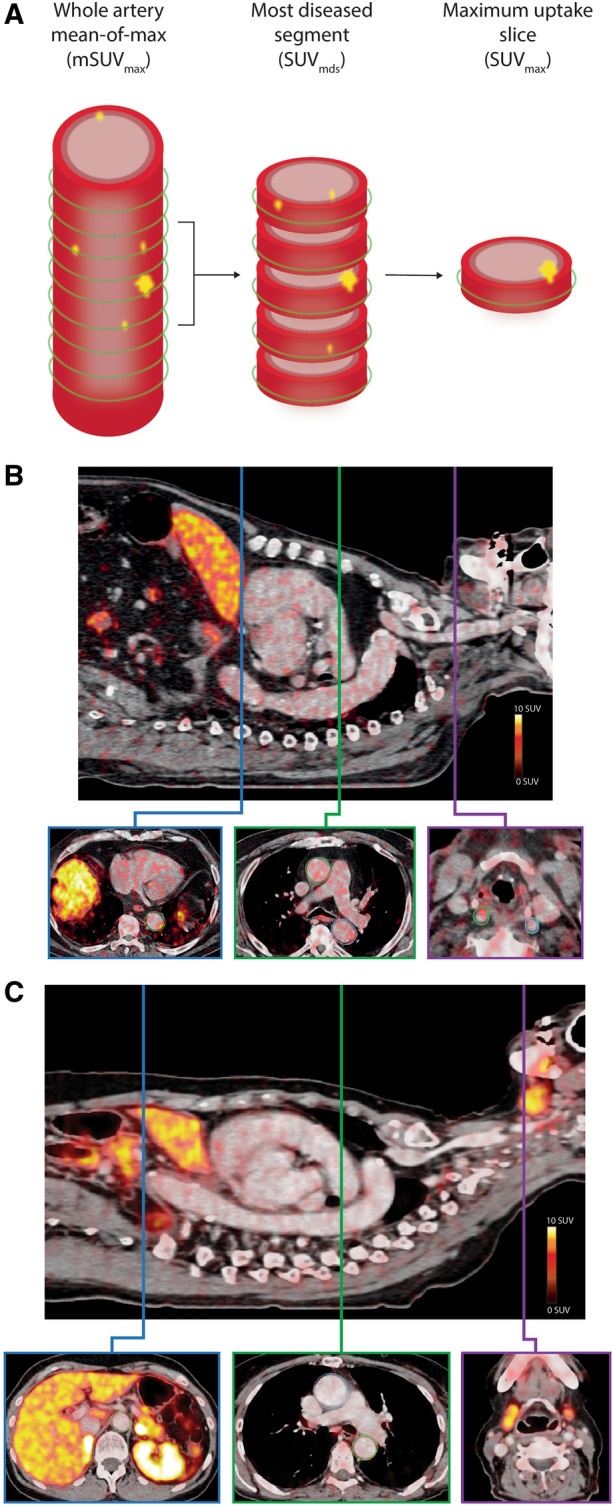
Vascular [^64^Cu]Cu-DOTATATE image analysis and uptake quantification. (**A**) Schematic illustration of an index vessel and measurement method of the 3 different uptake metrics. Green circle indicates region of interest placement. Examples of region of interest demarcation of the vessel wall shown on fused PET/CT images of a patient in the known CVD group (**B**) with vascular wall uptake of [^64^Cu]Cu-DOTATATE and a patient in the control group (**C**) with little [^64^Cu]Cu-DOTATATE uptake. Top images of both (**B**) and (**C**) are showing a sagittal slice for reference. Bottom images shows axial plane slices with drawn regions of interest of the ascending and descending aorta (blue and green boxes) and the carotid arteries (purple box)

All PET/CT scans were analyzed using the Osirix MD imaging software platform ver. 11.0 (Pixmeo, Bernex, Switzerland). ROI’s were drawn on axial slices of the aorta and the carotid arteries. ROI’s of the aorta were drawn on both the ascending and descending portions of the aorta. ROI’s were drawn from 1 slice cranial to the aortic valve to the branching of the right renal artery of the descending aorta. In the carotids, ROI’s were drawn from 5 slices cranial to the carotid bifurcation, on the internal carotid artery, and downward to the aorta or subclavian artery. Standardized uptake values (SUV) were measured from each ROI. To account for background [^64^Cu]Cu-DOTATATE uptake, SUV values were divided with a background SUV value measured as the mean of 4 adjacent slices in the right atrium, to allow for calculation of target-to-background (TBR) values. [^64^Cu]Cu-DOTATATE TBR and SUV uptake values are reported as whole-artery maximum (SUV_max_ and TBR_max_) and mean of max (mSUV_max_ and mTBR_max_), where max values from all slices were averaged. Also, a most-diseased segment (SUV_mds_ and TBR_mds_) of the artery approach, where the slice with the maximum SUV or TBR together with the 4 adjacent slices (2 on each side) was averaged, was also calculated and reported (Figure 2A). A representative example of the ROI’s drawn are shown in Figure 2B and C.

### Statistical analysis

Statistical analysis was performed using the programming language R version 4.1.2 (R Foundation, Vienna, Austria) with the integrated development environment RStudio version 2021.09.1 (RStudio, Boston, USA). Continuous normally distributed variables are reported as means with standard deviation (SD) or 95% confidence intervals (CI), and nonnormally distributed variables are reported as medians and interquartile range [IQR]. Differences in [^64^Cu]Cu-DOTATATE PET uptake values between the three groups were assessed using a linear multiple regression model with age, sex, smoking history, and BMI as co-variates and uptake value as dependent variable. Results of the linear multiple regression analyses are reported as parameter estimates of difference between the groups (Controls vs At-Risk group or known CVD group) with corresponding 95% CI and *P*-values, after adjustment of the aforementioned co-variates.

A random subset of ~ 25% (n = 20) of the total number PET/CT scans were assessed by a second trained reviewer to allow for assessment of agreement by calculation of the intraclass correlation coefficient (ICC) with 95% confidence intervals based on a single rating, consistency, two-way mixed effects model, and Bland–Altman plots. Reviewers were blinded to group allocation. ICC agreement was interpreted according to the standards previously proposed.^[20]^

Receiver operator characteristics (ROC) analysis were made using uptake values from the controls and the known CVD group, and area under the curve (AUC) was calculated along with optimal cut-point values using the point closest to (0,1) corner in the ROC plane approach with corresponding specificity and sensitivity percentages. Two-sided *P*-values < .05 were considered significant.

## Results

### Patient clinical characteristics

A total of 79 patients with a [^64^Cu]Cu-DOTATATE PET/CT scans available for analysis were divided into the 3 groups: 33 patients with at least 1 documented previous ischemic event, 24 patients with CVD risk factors but no ischemic event, and 22 controls without known CVD risk factors. Clinical characteristics of the 3 groups are listed in Table 1. All patients had a diagnosis of NEN. Patients in the known CVD group were in general more likely to have received a diagnosis of hyperlipidemia and type 2 diabetes compared to the At-Risk group.

**Table 1:** Clinical characteristics: Values are mean ± SD, n (%), or median [IQR]

	All (n = 79)	Known CVD (n = 33)	At-Risk (n = 24)	Controls (n = 22)
Age at scan—years	68 (10.2)	72 (9.3)	66 (9.8)	65 (10.5)
Sex—female (%)	35 (44%)	13 (39%)	11 (46%)	11 (50%)
Body mass index at scan (kg/m2)	26.7 (5.0)	25.7 (3.7)	29.4 (5.9)	25.2 (4.8)
Smoking history (%)	42 (53%)	20 (61%)	13 (54%)	6 (27%)
Type 2 diabetes	16 (20%)	10 (30%)	6 (25%)	0 (0%)
Hypertension	45 (57%)	27 (82%)	21 (88%)	0 (0%)
Hyperlipidemia	48 (61%)	29 (88%)	16 (67%)	0 (0%)
Lipid lowering medication	41 (52%)	28 (85%)	13 (54%)	0 (0%)
Event type				
TIA/ischemic stroke	21 (27%)	21 (64%)	0 (0%)	0 (0%)
Myocardial infarction	12 (15%)	12 (36%)	0 (0%)	0 (0%)
Anticoagulation therapy				
NOAC	7 (9%)	5 (15%)	2 (9%)	0 (0%)
Aspirin	12 (15%)	9 (27%)	3 (13%)	0 (0%)
ADP inhibitor	16 (20%)	16 (49%)	0 (0%)	0 (0%)
NEN type				
Small intestine	35 (44.3%)	19 (57.6%)	5 (20.8%)	11 (50%)
Colon	5 (6.3%)	2 (6.1%)	2 (8.3%)	1 (4.5%)
Lung	8 (10.1%)	4 (12.1%)	3 (12.5%)	1 (4.5%)
Pancreas	21 (26.6%)	6 (18.2%)	11 (45.8%)	4 (18.2%)
Other/unknown	10 (12.7%)	2 (6.1%)	3 (12.5%)	5 (22.7%)
Age at ischemic event		68 (9.5)	–	–
Median time from event to scan (months)	–	37 [16–64]	–	–
Median % 10-year Framingham risk score		–	15 [11–30]	–

*ADP*, adenosine diphosphate; *CVD*, cardiovascular disease; *NEN*, neuroendocrine neoplasm; *NOAC*, non-vitamin K oral anticoagulants; *TIA*, transient ischemic attack

### [^64^Cu]Cu-DOTATATE uptake of the carotid arteries measured as SUV

All unadjusted mean differences between the groups along with parameter estimates for the group differences from the multiple regression analyses are listed in Table 2.

**Table 2:** Unadjusted mean differences between groups and results from the multiple regression analyses with sex, BMI, smoking history, and age as co-variates and either aorta or carotid uptake values as the dependent variable

	Mean difference	Estimate (*β*)	95% CI	*P*-value (*β*)
Carotid				
SUV_max_				
Controls vs At-Risk	0.29	0.02	[− 0.55 to 0.59]	.94
Controls vs Known CVD	0.59	0.60	[0.05 to 1.15]	.03*
At-Risk vs Known CVD	0.30	0.58	[0.07 to 1.09]	.02*
mSUV_max_				
Controls vs At-Risk	0.05	0.01	[− 0.19 to 0.21]	.92
Controls vs Known CVD	0.10	0.09	[− 0.10 to 0.28]	.35
At-Risk vs Known CVD	0.05	0.08	[− 0.10 to 0.26]	.37
SUV_mds_				
Controls vs At-Risk	0.10	< 0.01	[− 0.37 to 0.38]	.99
Controls vs Known CVD	0.34	0.36	[0.004 to 0.72]	.05*
At-Risk vs Known CVD	0.24	0.37	[0.02 to 0.69]	.03*
TBR_max_				
Controls vs At-Risk	0.91	0.52	[− 0.41 to 1.45]	.27
Controls vs Known CVD	1.22	1.24	[0.35 to 2.14]	.007*
At-Risk vs Known CVD	0.31	0.73	[− 0.10 to 1.56]	.09
mTBR_max_				
Controls vs At-Risk	0.30	0.24	[− 0.11 to 0.58]	.18
Controls vs Known CVD	0.93	0.42	[0.09 to 0.76]	.01*
At-Risk vs Known CVD	0.09	0.19	[− 0.12 to 0.50]	.23
TBR_mds_				
Controls vs At-Risk	0.43	0.27	[− 0.31 to 0.85]	.35
Controls vs Known CVD	0.71	0.77	[0.21 to 1.33]	.008*
At-Risk vs Known CVD	0.28	0.50	[− 0.02 to 1.01]	.06
Aorta				
SUV_max_				
Controls vs At-Risk	0.40	− 0.20	[− 1.07 to 0.68]	.66
Controls vs Known CVD	0.61	0.18	[− 0.66 to 1.03]	.67
At-Risk vs Known CVD	0.21	0.38	[− 0.41 to 1.16]	.34
mSUV_max_				
Controls vs At-Risk	0.020	− 0.15	[− 0.45 to 0.15]	.33
Controls vs Known CVD	0.034	− 0.10	[− 0.39 to 0.19]	.48
At-Risk vs Known CVD	0.014	0.04	[− 0.23 to 0.31]	.75
SUV_mds_				
Controls vs At-Risk	0.17	− 0.08	[− 0.61 to 0.44]	.75
Controls vs Known CVD	0.32	0.15	[− 0.36 to 0.65]	.56
At-Risk vs Known CVD	0.15	0.23	[− 0.24 to 0.70]	.33
TBR_max_				
Controls vs At-Risk	1.35	0.47	[− 0.78 to 1.73]	.46
Controls vs Known CVD	1.47	1.04	[− 0.17 to 2.25]	.09
At-Risk vs Known CVD	0.11	0.56	[− 0.56 to 1.69]	.32
mTBR_max_				
Controls vs At-Risk	0.41	0.17	[− 0.27 to 0.61]	.45
Controls vs Known CVD	0.44	0.32	[− 0.11 to 0.74]	.14
At-Risk vs Known CVD	0.02	0.15	[− 0.24 to 0.54]	.45
TBR_mds_				
Controls vs At-Risk	0.69	0.29	[− 0.47 to 1.05]	.45
Controls vs Known CVD	0.90	0.77	[0.03 to 1.50]	.04*
At-Risk vs Known CVD	0.22	0.48	[− 0.21 to 1.16]	.17

Estimates (β) are the difference between the groups listed when controlling for the co-variates. *P*-values reported in the table are for the estimates

**Figure 3:**
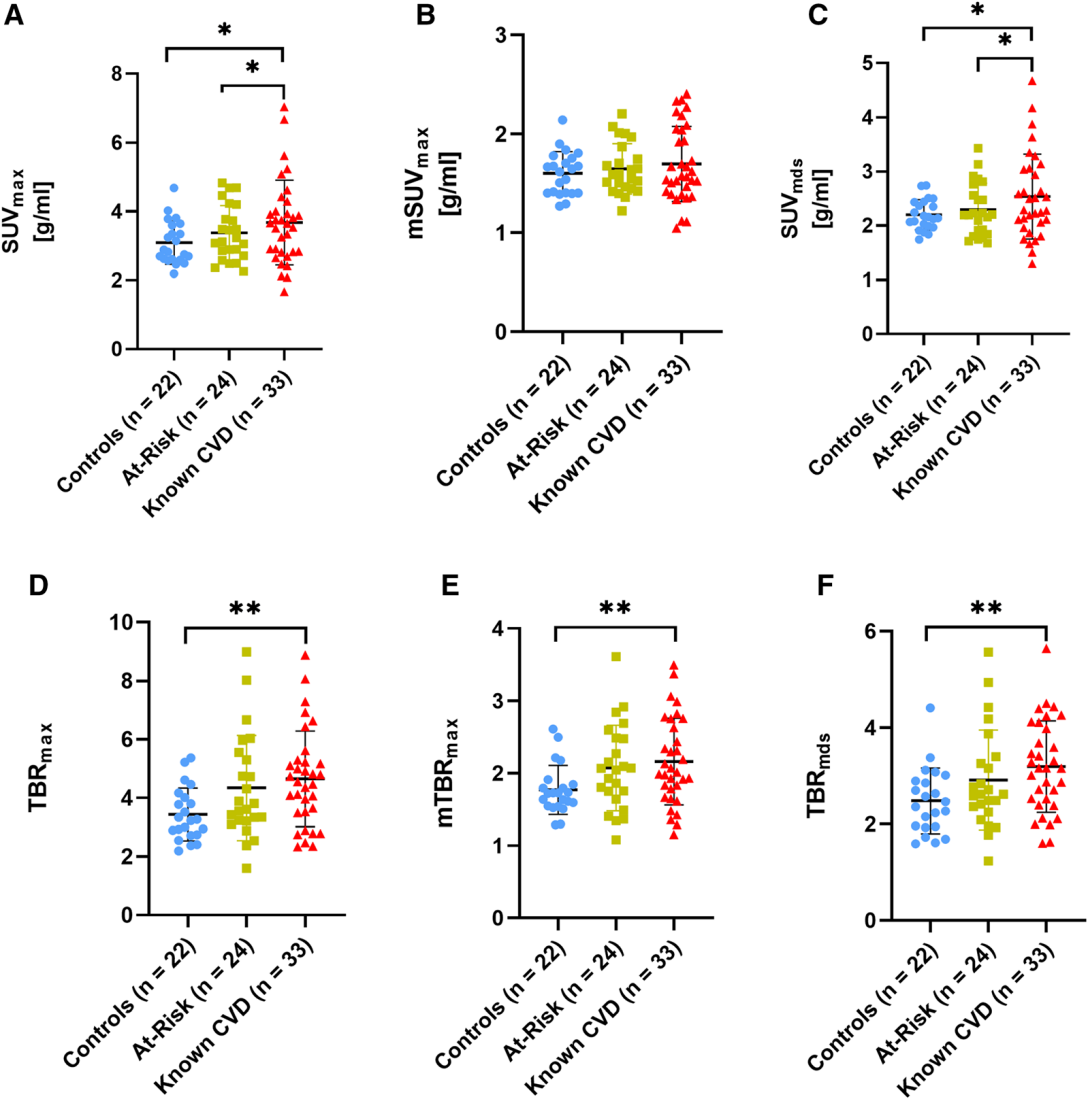
Difference between groups in carotid [^64^Cu]Cu-DOTATATE PET/CT uptake metrics was more pronounced compared to the aorta. Scatter plots showing both SUV (**A**-**C**) and TBR (**D**-**F**) uptake metrics of the aorta, visualized as means ± SD of the 3 groups. Analyses of uptake metrics, after adjustment of co-variates, in carotid arteries showed a better ability to discriminate between groups compared to the analyses of the aorta. SUVmax (*P* = .03), SUVmds (*P* = .05), TBRmax (*P* < .01), mTBRmax (*P* = .01), and TBRmds (*P* < .01) all showed a significant difference between the controls and known CVD group. No difference was observed with regards to group difference in mSUVmax. The SUVmax (*P* = .02) and SUVmds (*P* = .03) were also different between the At-Risk and known CVD groups. *Mds*, most-diseased segment; *SUV*, standardized uptake value; *TBR*, target-to-background ratio

PET uptake, measured as SUV metrics, in the carotid arteries showed a significant difference in SUV_max_ between the controls and the known CVD group and the At-Risk vs known CVD group, but not between the controls and At-Risk group (controls: 3.09 ± 0.63; At-Risk: 3.38 ± 0.79; known CVD: 3.68 ± 1.23) (Figure 3A; Table 2). Similarly, when measuring SUV_mds_ (controls: 2.20 ± 0.28; At-Risk: 2.30 ± 0.49; known CVD: 2.54 ± 0.78) differences were observed between the controls and known CVD group and the At-Risk and known CVD groups, but not the controls and At-Risk group (Figure 3C; Table 2). Measurements of mSUV_max_ (controls: 1.60 ± 0.22; At-Risk: 1.65 ± 0.25; known CVD: 1.70 ± 0.38) displayed no differences between the groups (Figure 3B; Table 2). Unadjusted group means for all uptake values are also listed in Supplemental Table I.

**Figure 4:**
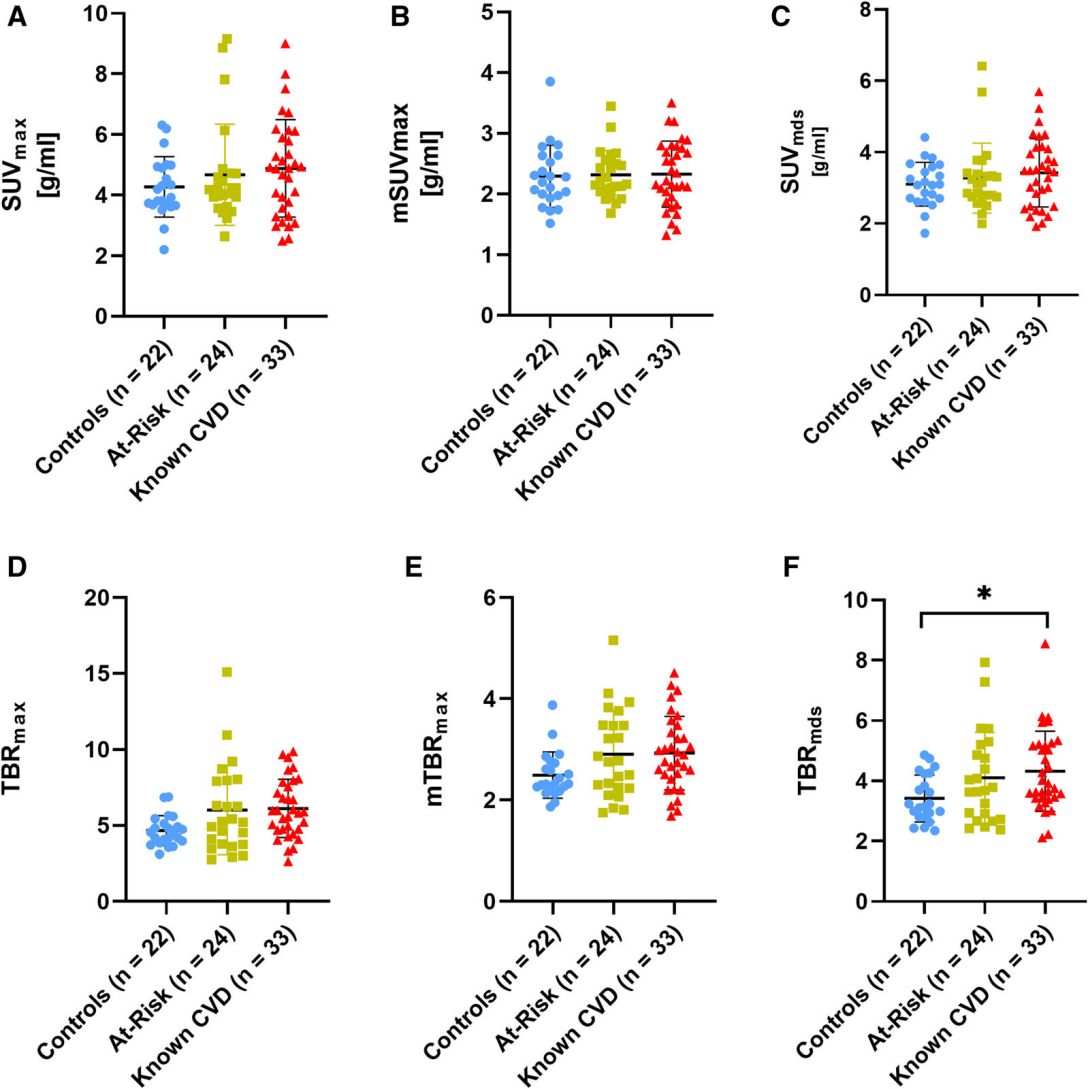
Aortic [^64^Cu]Cu-DOTATATE PET/CT uptake metrics. Scatter plots showing both SUV (**A**-**C**) and TBR (**D**-**F**) uptake metrics of the aorta, with means ± SD of the 3 groups. After adjustment of co-variates (age, sex, BMI, and smoking history) in the multivariate model, TBRmds was the only uptake metric observed to be significantly different between the controls and known CVD group (*P* = .04). *CVD*, cardiovascular disease; *CT*, computed tomography; *NEN*, neuroendocrine neoplasia; *PET*, positon emission tomography; *Mds*, most-diseased segment; *SUV*, standardized uptake value; *TBR*, target-to-background ratio

### [^64^Cu]Cu-DOTATATE uptake of the carotid arteries measured as TBR

Venous SUV background measures for calculation of TBR metrics were comparable between the groups (controls vs At-Risk: − 0.08, 95% CI [− 0.27 to 0.09], *P* = .34; controls vs known CVD: − 0.13, 95% CI [− 0.31 to 0.05], *P* = .15; At-Risk vs known CVD: − 0.04, 95% CI [− 0.21 to 0.12], *P* = .60).

Differences in TBR uptake metrics between the groups for the carotid arteries were observed between the controls and known CVD group with regards to both TBR_max_ (controls: 3.43 ± 0.90; At-Risk: 4.34 ± 1.80; known CVD: 4.66 ± 1.63), mTBR_max_ (controls: 1.77 ± 0.33; At-Risk: 2.07 ± 0.59; known CVD: 2.16 ± 0.60), and TBR_mds_ (controls: 3.41 ± 0.78; At-Risk: 4.10 ± 1.51; known CVD: 4.32 ± 1.33) (Figure 3D-F; Table 2). No significant differences were observed when comparing the controls and At-Risk group, with regards to any of the uptake metrics. Trends toward significance were observed when comparing the At-Risk and known CVD groups with regards to TBR_max_.

### [^64^Cu]Cu-DOTATATE uptake of the aorta measured as SUV

Compared to uptake values from the carotids, values obtained from the aorta were largely observed to have higher values (Figures 3, 4).

[^64^Cu]Cu-DOTATATE uptake quantified by SUV in the aorta was generally observed to be higher in patients with known CVD compared especially to control patients and less compared to those At-Risk of CVD. Differences between groups were largest for SUV_max_ (controls: 4.27 ± 1.00; At-Risk: 4.67 ± 1.68; known CVD: 4.88 ± 1.61) and SUV_mds_ (controls: 3.10 ± 0.62; At-Risk: 3.27 ± 0.98; known CVD: 3.42 ± 0.95) and negligible for the whole-artery mSUV_max_ (controls: 2.29 ± 0.51; At-Risk: 2.31 ± 0.40; known CVD: 2.33 ± 0.54) (Figure 4A-C; Table 2). We found no significant differences between any of the groups in the multivariate analyses, neither when assessing the whole-artery mSUV_max_ localized nor SUV_mds_ measured in the aorta (Table 2).

### [^64^Cu]Cu-DOTATATE uptake of the aorta measured as TBR

When calculating TBR uptake metrics differences between the groups were more evident than when using SUV metrics (Fig. 4 D-F). A significant difference was observed between the controls and Known CVD group with regards to TBR_mds_, while no difference was observed when comparing the controls with the At-Risk group and the At-Risk with the known CVD group (controls: 3.42 ± 0.78; At-Risk: 4.10 ± 1.51; known CVD: 4.32 ± 1.33). TBR_max_ (controls: 4.65 ± 0.98; At-Risk: 6.00 ± 2.93; known CVD: 6.12 ± 1.92) and mTBR_max_ (controls: 2.49 ± 0.46; At-Risk: 2.91 ± 0.87; known CVD: 2.93 ± 0.73) of the aorta did not show a significant difference between any of the groups (Table 2).

### Reproducibility

**Figure 5:**
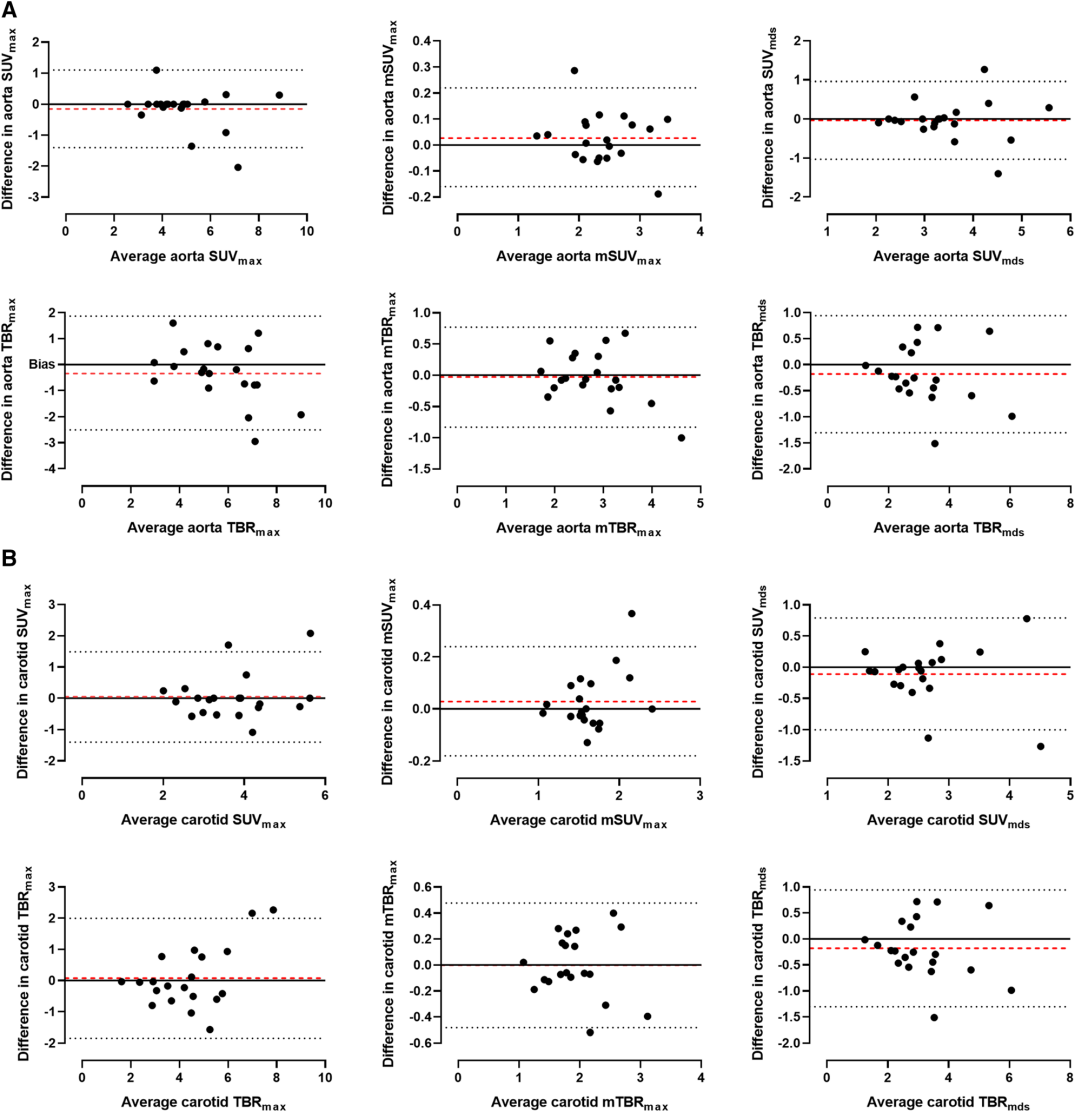
Bland–Altman plots of measurements performed by the 2 reviewers with fixed bias shown as red-dotted lines. **A** Aortic and **B** carotid measures

The reproducibility (ICC) of the [^64^Cu]Cu-DOTATATE SUV measurements, of both the aorta and carotid arteries, between the 2 reviewers were excellent (Aorta: 0.97; 95%CI [0.96 to 0.98] and Carotids: 0.94; 95% CI [0.92 to 0.96]). Likewise, when measuring the TBR values, ICC of the aortic and carotid measurements were good and excellent, respectively (Aorta: 0.89; 95% CI [0.83 to 0.93] and carotids: 0.91; 95% CI [0.87 to 0.94]). Bland–Altman plots for both aorta and carotids regarding all uptake metrics are shown in Figure 5. Here, only small fixed and proportional biases were observed between the reviewers.

### Optimal cut-point values

**Figure 6:**
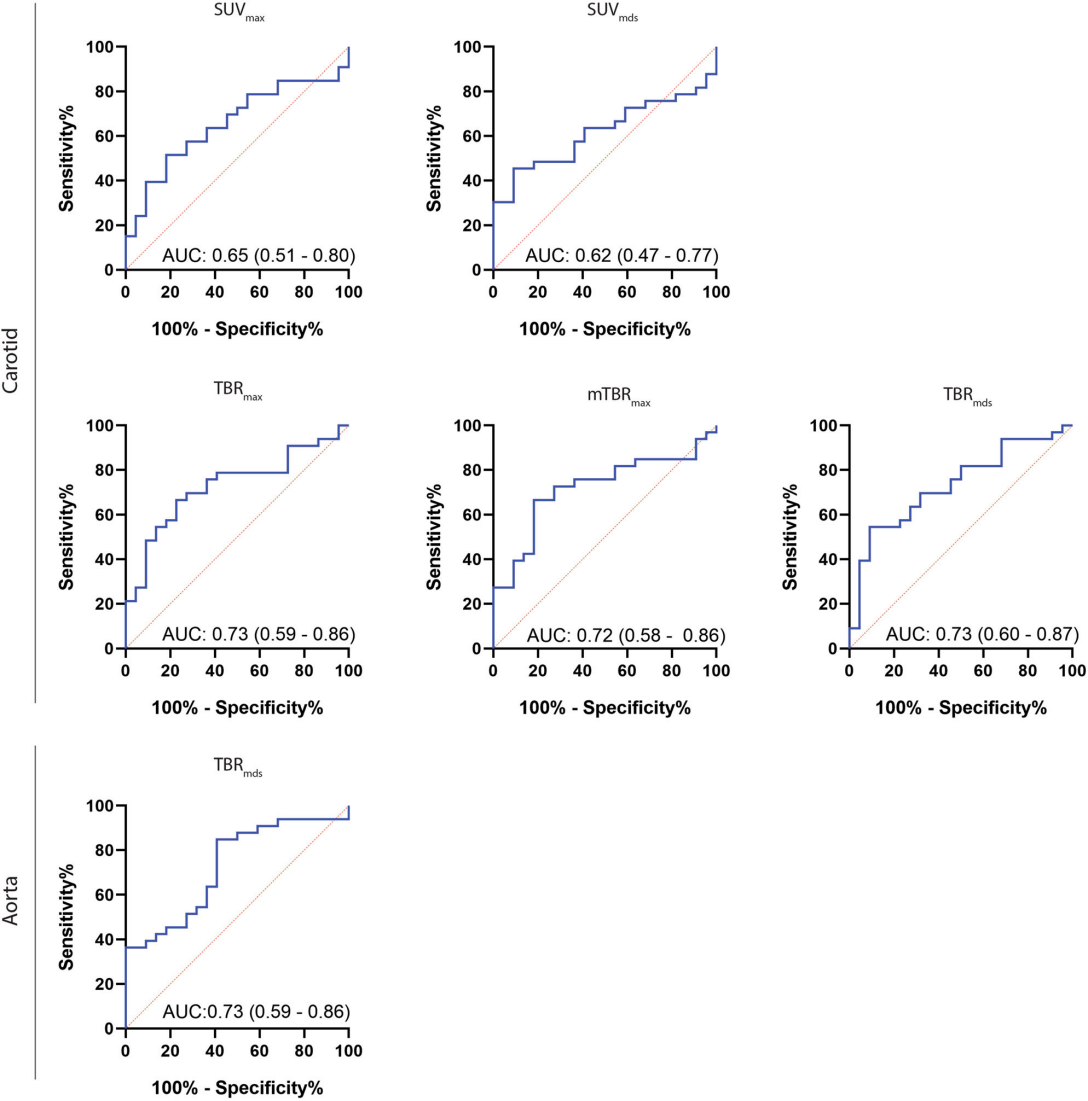
Receiver operating characteristic curves with AUC estimates and corresponding 95% confidence intervals between the controls and known CVD groups for measures where a significant difference was observed. Best AUC measures were observed for the TBR metrics. *AUC*, area under the curve; *CVD*, cardiovascular disease; *CT*, computed tomography; *NEN*, neuroendocrine neoplasia; *PET*, positon emission tomography; *Mds*, most-diseased segment; *SUV*, standardized uptake value; *TBR*, target-to-background ratio

As an exploratory analysis, ROC curves were made for the PET uptake values that were observed to be significantly different between the controls and known CVD group in the multiple regression analysis. The area under the curve (AUC) for the TBR values were all similar (~ 0.73) as seen in Figure 6. AUCs for the significant SUV metrics were lower. Optimal cut-point values and corresponding specificity and sensitivity estimates are listed in Table 3. In line with the AUC measurements, greater specificity and sensitivity were achieved with the TBR metrics compared to SUV.

**Table 3:** Optimal cut-point values for predicting no CVD vs CVD with corresponding sensitivity and specificity percentages

	Cut-point value	Sensitivity (%)	95% CI	Specificity (%)	95% CI
Carotid					
SUV_max_	3.43	58	[41 to 73]	73	[52 to 87]
SUV_mds_	2.47	48	[33 to 65]	82	[61 to 0.93]
TBR_max_	4.04	67	[50 to 80]	77	[57 to 90]
mTBR_max_	1.92	67	[50 to 80]	81	[61 to 93]
TBR_mds_	2.70	70	[53 to 83]	68	[47 to 84]
Aorta					
TBR_mds_	3.26	85	[69 to 93]	59	[39 to 75]

## Discussion

Visualization and quantification of arterial atherosclerosis using multimodality PET have been investigated for the past 20 years, starting with the first reports using [^18^F]FDG as the tracer of choice.^[21,22]^ Since then, both improvements in imaging methods and technological advancement have made PET more readily available, and novel radiotracers offer promise of more specific imaging of the physiological processes of atherosclerosis.^[23]^

In the present retrospective study, we observed a gradual increase in arterial uptake of [^64^Cu]Cu-DOTATATE from control patients without known CVD to patients at-risk and to patients with known CVD. The difference in carotid uptake between healthy controls and patients with known CVD was significant. The aortic uptake was higher than the carotid uptake, but with larger variance leading to mostly non-significant differences in the aorta between the groups.

### Methods of tracer uptake quantification

Several methods have been used to quantify [^18^F]FDG uptake in previous studies. In the present study, we sought to investigate these methods when imaging atherosclerosis using the radiolabeled tracer [^64^Cu]Cu-DOTATATE in NEN patients with different degrees of CVD compared to controls with no known cardiovascular risk factors. PET [^64^Cu]Cu-DOTATATE imaging has previously been used to study atherosclerosis, but this is the first time that it has been used to investigate potential differences between retrospectively pre-defined groups with varying degrees of CVD.

When comparing uptake between the groups using the carotid arteries as the vessel of interest, significant differences between the controls and the known CVD group were observed for all uptake metrics except mSUV_max_ (Table 2). Comparisons between the at-risk and the known CVD group showed significant differences with regards to SUV_max_ and SUV_mds_.

The only uptake metric that showed a group-wise difference when using the aorta as the vessel of interest was TBR_mds_. None of the other uptake metrics measured in the aorta showed any difference as seen in Figure 4 and Table 2. It is worth noticing that in this study, a large portion of the aorta was quantified (both ascending and descending), compared to other studies using [^18^F]FDG that measured a smaller segment.^[8,24]^ Analyzing the ascending aorta alone did not change these conclusions (Supplemental Figure I).

A variety of uptake metrics and vessels of interest have been used in other studies, and guidelines as to which metrics and vessels to image for quantification of atherosclerosis have been published for [^18^F]FDG-related imaging.^[8,25]^ Similar recommendations do not exist for [^64^Cu]Cu-DOTATATE imaging. Based on our results, TBR_max_ or TBR_mds_ measures in the carotid artery seem to be the most robust method for quantifying in vivo uptake of [^64^Cu]Cu-DOTATATE and detect differences in subjects with varying degrees of CVD compared to the mean-of-max approach. This point of view was further corroborated by ROC curve analyses, where TBR_max_ and TBR_mds_ yielded the best results with respect to cut-point values and corresponding sensitivity and specificity. This is equivalent to the results previously found with [^18^F]FDG.^[8]^

It can be speculated that the differentiation between patients with or without CVD might have been improved in a prospective set-up, which would have made group allocation less prone to selection bias. In addition, allocation to the at-risk group in the present study was determined by the presence of one or more pre-defined risk factors for CVD, whereas a previous similar study investigating [^18^F]FDG allocated on the basis of two or more risk factors.^[8]^

### Comparison to in vivo imaging using other PET tracers

[^18^F]FDG is the most thoroughly investigated radiolabeled tracer used for PET imaging in atherosclerosis, has shown the ability to detect differences in uptake between groups with varying degrees of CVD, and proven a valuable tool in detecting the effects of various drugs in relation to atherosclerosis.^[8,9,24]^ Other tracers, in particular [^18^F]-sodium fluoride primarily related to micro calcification, have also been heavily investigated in the context of atherosclerosis and have shown promising results.^[26]^ [^64^Cu]Cu-DOTATATE on the other hand offers the possibility of directly imaging-activated macrophages in atherosclerosis enabling a more specific approach as opposed to [^18^F]FDG.^[15,27,28]^ The majority of research in the field of atherosclerotic PET imaging using SST_2_ ligand tracers has been done with ^68^Ga-labeled DOTATATE, and some studies have found correlations between [^68^Ga]Ga-DOTATATE PET uptake measures and CVD risk factors.^[14,29]^

Studies using ^64^Cu-labeled DOTATATE for PET imaging of atherosclerosis are sparse but as with ^68^Ga evidence suggests that uptake correlates with established risk factors of CVD.^[11,16,30]^

DOTATATE has also been shown to be a marker of specific macrophage activity in studies using both histological and cell-specific assays, where it also outperformed [^18^F]FDG as a marker of coronary inflammation.^[12]^ Although, some early ex vivo studies did find low expression of the SST_2_ receptor on endothelial cells.^[31,32]^ SST signaling in vascular tissue cells has been shown to serve an anti-inflammatory function, but the importance of this in relation to atherosclerosis and SST imaging has not yet been established.^[33]^

[^64^Cu]Cu-DOTATATE also has the distinct advantage of a shorter positron range (~ 1 mm) as opposed to its ^68^Ga-labeled counterpart (~ 4 mm). This offers better spatial resolution, which is of paramount importance when imaging smaller structures as arterial vessel walls, including coronary arteries, which has also been shown to be feasible using [^64^Cu]Cu-DOTATATE in patients with type 2 diabetes.^]13,34]^

As previously mentioned, another study investigated the performance and optimal metrics using [^18^F]FDG uptake as a marker of atherosclerotic inflammation. Compared to this, [^64^Cu]Cu-DOTATATE uptake might seem to have underperformed somewhat in the ability to discriminate between subjects with and without established CVD compared to [^18^F]FDG.^[8]^ However, important differences in study design should be considered, as the present study is retrospective in its nature.

Another aspect that should be considered is the effective radiation dose observed when using the different radiotracers. The clinical [^64^Cu]Cu-DOTATATE PET used in the present study exposes the patients to approximately 6-7 mSv.^[35]^ Because of methodological differences, the effective dose in atherosclerosis studies using [^18^F]FDG varies quite considerably between 4 and 7 mSv.^[6,8,9,36]^ [^68^Ga]Ga-DOTATATE exposes patients to less radiation, with an effective dose of 3-5 mSv.^[35,37]^ Hopefully, technological advances in scanner and image reconstruction technology will allow for PET imaging with lower radiation doses. This is also a prerequisite if atherosclerosis imaging using PET is to become a routine clinical examination.

### Reproducibility

The results from the present study are in line with previous investigations that have shown the reproducibility of PET measurements in atherosclerosis to be excellent.^[8,38]^ We did observe that the ICC was somewhat lower for the TBR measurements, probably due to the dependency of two measurements, as opposed to only one for the SUV’s, which should be considered when designing trials using [^64^Cu]Cu-DOTATATE TBR metrics as endpoints. No fixed or proportional bias was observed in the Bland–Altman analysis between the two reviewers, which testifies to the robustness of the methods. The contrast-enhanced PET/CT scans used in this study allow for good anatomical discrimination of the aorta and carotid arteries, but is not the standard when comparing to similar studies that most often use low-dose non-contrast-enhanced CT scans.^[8,13,39]^ This might limit the generalizability of the results.

### Limitations

Some limitations apply to the present study. Firstly, this is a retrospective study and all data were collected from electronic medical records, which makes it prone to selection bias. Furthermore, we did not assess any data beyond what was documented in the electronic medical records. Therefore, no predictive inferences can be drawn from it. Secondly, the patients included in this study were all diagnosed with NEN and received various treatments for this, which might have affected tracer uptake and limits the generalizability of the results to a broader population. It would have been a meaningful addition to evaluate the uptake of the coronary arteries between the groups, but the routine clinical PET/CT scan protocol used in this cohort of patients did not allow for coronary measurement due to lack of gating of respiratory and cardiac movement.

## Conclusion

In the present study, we were able to demonstrate differences in some of the most frequently reported PET uptake metrics using [^64^Cu]Cu-DOTATATE as a marker of atherosclerotic inflammation in patients with different degrees of known CVD. Measurements of the carotid artery as either maximum uptake values or most-diseased segment analysis showed the best ability to discriminate between the groups. The present study paves the way for further prospective investigations to validate and establish the value of [^64^Cu]Cu-DOTATATE PET as a method for imaging and quantifying macrophage burden and therefore inflammation in atherosclerosis.

## New knowledge gained

PET uptake measurements of the activated macrophage tracer [^64^Cu]Cu-DOTATATE are reproducible and able to demonstrate differences in vascular inflammation between groups with different degrees of CVD when using the most frequently reported uptake metrics.

## Supplementary Information

Below is the link to the electronic supplementary material.

Supplementary file1 (DOCX 12 kb)

Supplementary file2 (PPTX 540 kb)
