# Supplementary material for: [64Cu]Cu-DOTATATE PET metrics in the investigation of atherosclerotic inflammation in humans
Source: J Nucl Cardiol. 2022 Aug 31;30(3):986–1000. doi: 10.1007/s12350-022-03084-4 (PMC10261263; doi:10.1007/s12350-022-03084-4)
Supplement: Supplementary file 1 — Supplementary file1 (DOCX 12 kb) [file 12350_2022_3084_MOESM1_ESM.docx]

**Abbreviations**

CT – computed tomography

CVD – cardiovascular disease

DOTATATE - [1,4,7,10-tetraazacyclododecane-N,N’, N’’,N’’’-tetraacetic acid]-D-Phe1, Tyr3-octreotate

EMR – electronic medical record

FDG - fluoro-deoxyglucose

NEN – neuroendocrine neoplasm

PET – positron emission tomography

SST_2_ - somatostatin receptor subtype-2

SUV – standardized uptake value

TBR – target-to-background
