# Supplementary material for: [64Cu]Cu-DOTATATE PET metrics in the investigation of atherosclerotic inflammation in humans
Source: J Nucl Cardiol. 2022 Aug 31;30(3):986–1000. doi: 10.1007/s12350-022-03084-4 (PMC10261263; doi:10.1007/s12350-022-03084-4)
Supplement: Supplementary file 2 — Supplementary file2 (PPTX 540 kb) [file 12350_2022_3084_MOESM2_ESM.pptx]

## Slide 1
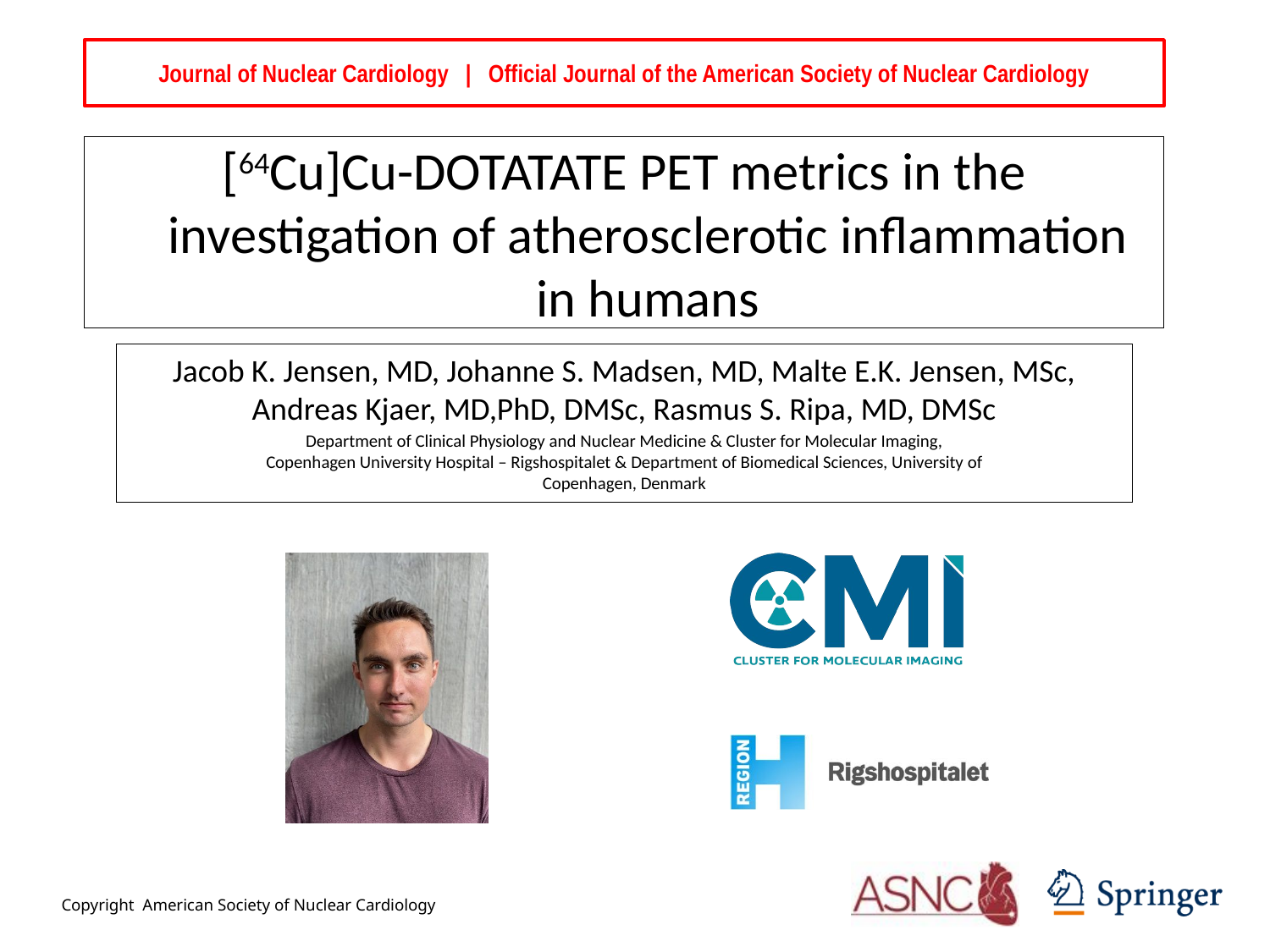

Journal of Nuclear Cardiology | Official Journal of the American Society of Nuclear Cardiology
# [64Cu]Cu-DOTATATE PET metrics in the investigation of atherosclerotic inflammation in humans
Jacob K. Jensen, MD, Johanne S. Madsen, MD, Malte E.K. Jensen, MSc, Andreas Kjaer, MD,PhD, DMSc, Rasmus S. Ripa, MD, DMSc
Department of Clinical Physiology and Nuclear Medicine & Cluster for Molecular Imaging,
Copenhagen University Hospital – Rigshospitalet & Department of Biomedical Sciences, University of
Copenhagen, Denmark
Copyright American Society of Nuclear Cardiology

## Slide 2
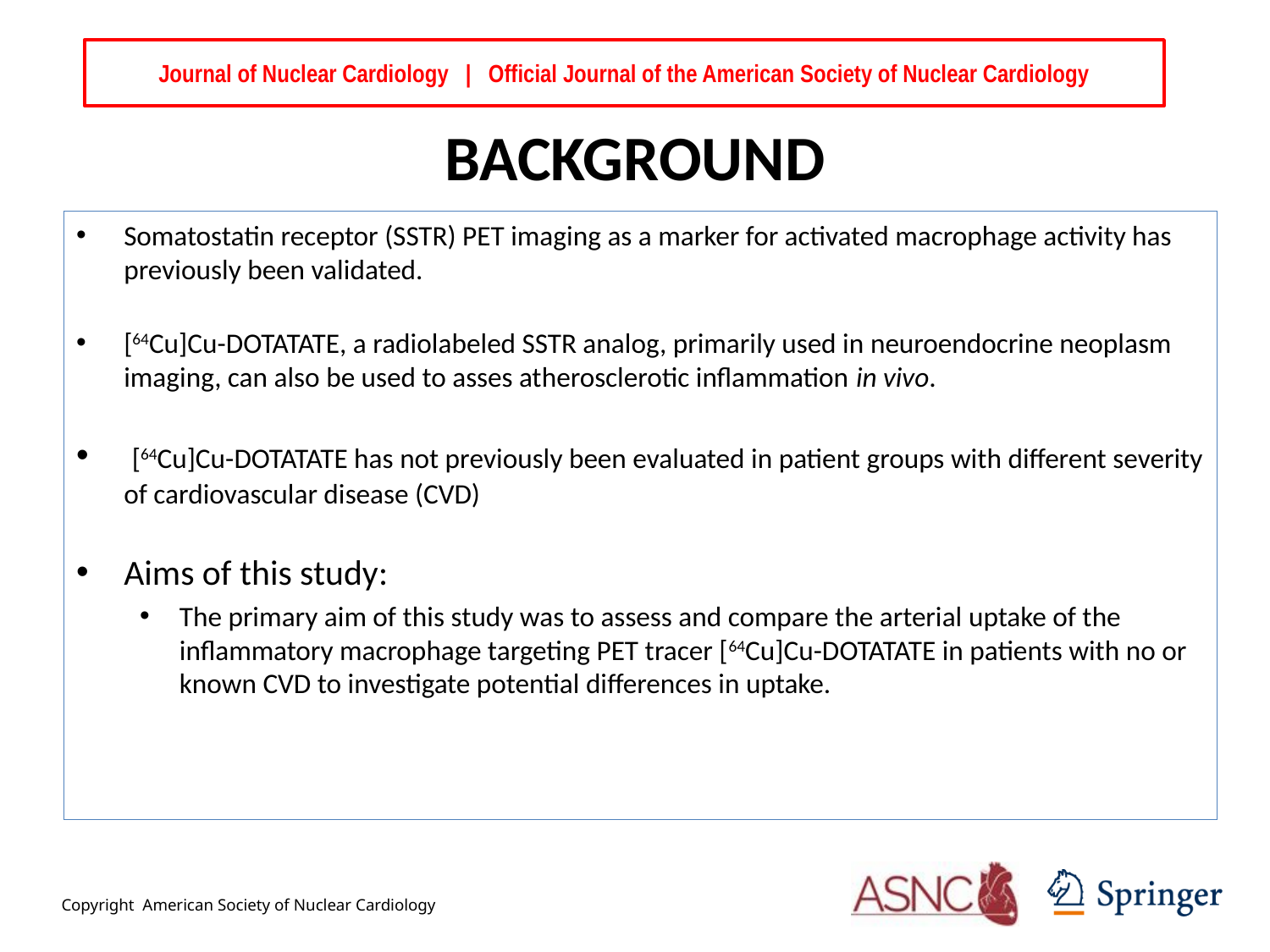

Journal of Nuclear Cardiology | Official Journal of the American Society of Nuclear Cardiology
# BACKGROUND
Somatostatin receptor (SSTR) PET imaging as a marker for activated macrophage activity has previously been validated.
[64Cu]Cu-DOTATATE, a radiolabeled SSTR analog, primarily used in neuroendocrine neoplasm imaging, can also be used to asses atherosclerotic inflammation in vivo.
 [64Cu]Cu-DOTATATE has not previously been evaluated in patient groups with different severity of cardiovascular disease (CVD)
Aims of this study:
The primary aim of this study was to assess and compare the arterial uptake of the inflammatory macrophage targeting PET tracer [64Cu]Cu-DOTATATE in patients with no or known CVD to investigate potential differences in uptake.
Copyright American Society of Nuclear Cardiology

## Slide 3
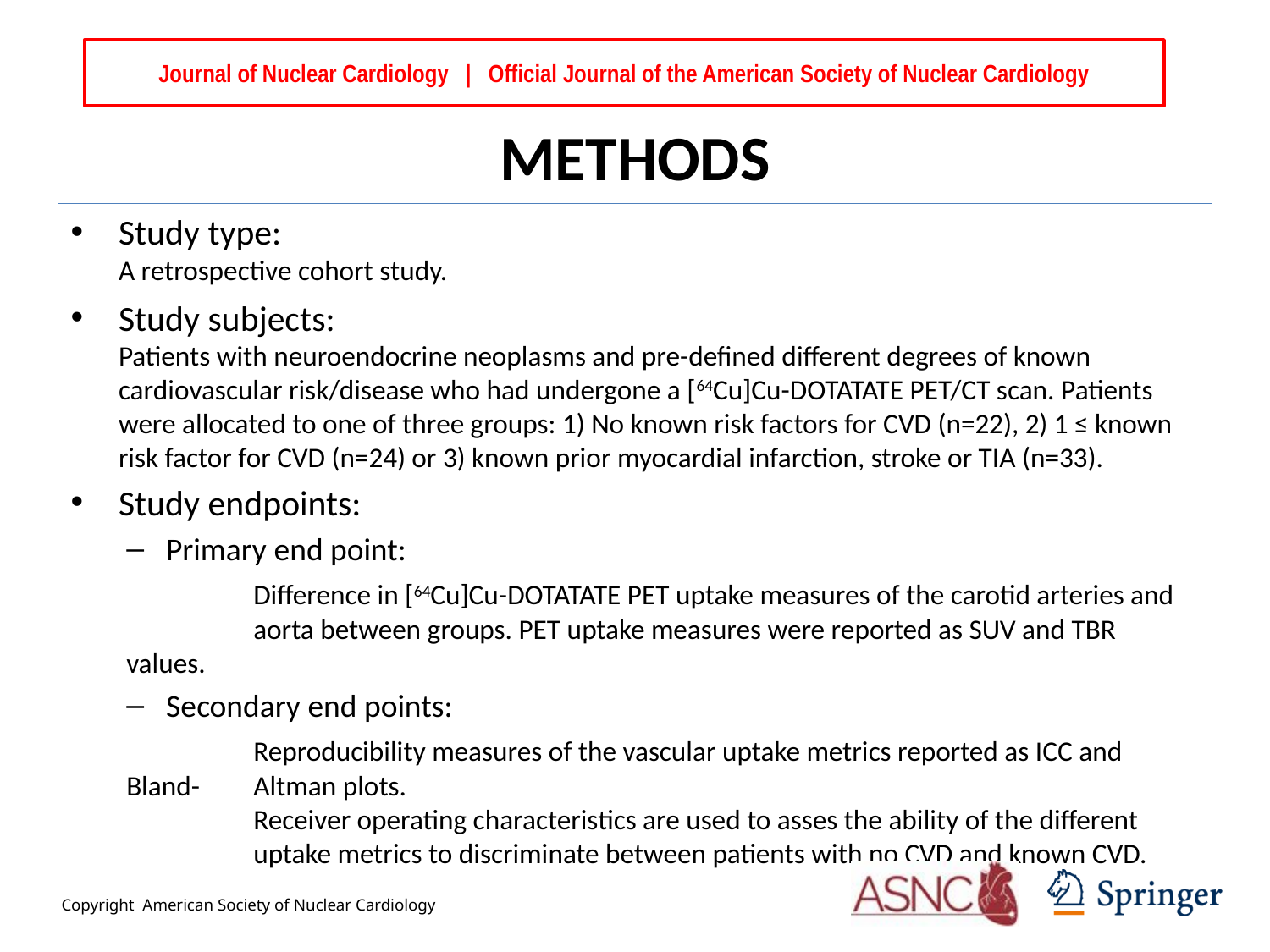

Journal of Nuclear Cardiology | Official Journal of the American Society of Nuclear Cardiology
# METHODS
Study type:
A retrospective cohort study.
Study subjects:
Patients with neuroendocrine neoplasms and pre-defined different degrees of known cardiovascular risk/disease who had undergone a [64Cu]Cu-DOTATATE PET/CT scan. Patients were allocated to one of three groups: 1) No known risk factors for CVD (n=22), 2) 1 ≤ known risk factor for CVD (n=24) or 3) known prior myocardial infarction, stroke or TIA (n=33).
Study endpoints:
Primary end point:
	Difference in [64Cu]Cu-DOTATATE PET uptake measures of the carotid arteries and 	aorta between groups. PET uptake measures were reported as SUV and TBR values.
Secondary end points:
	Reproducibility measures of the vascular uptake metrics reported as ICC and Bland-	Altman plots.
	Receiver operating characteristics are used to asses the ability of the different 	uptake metrics to discriminate between patients with no CVD and known CVD.
Copyright American Society of Nuclear Cardiology

## Slide 4
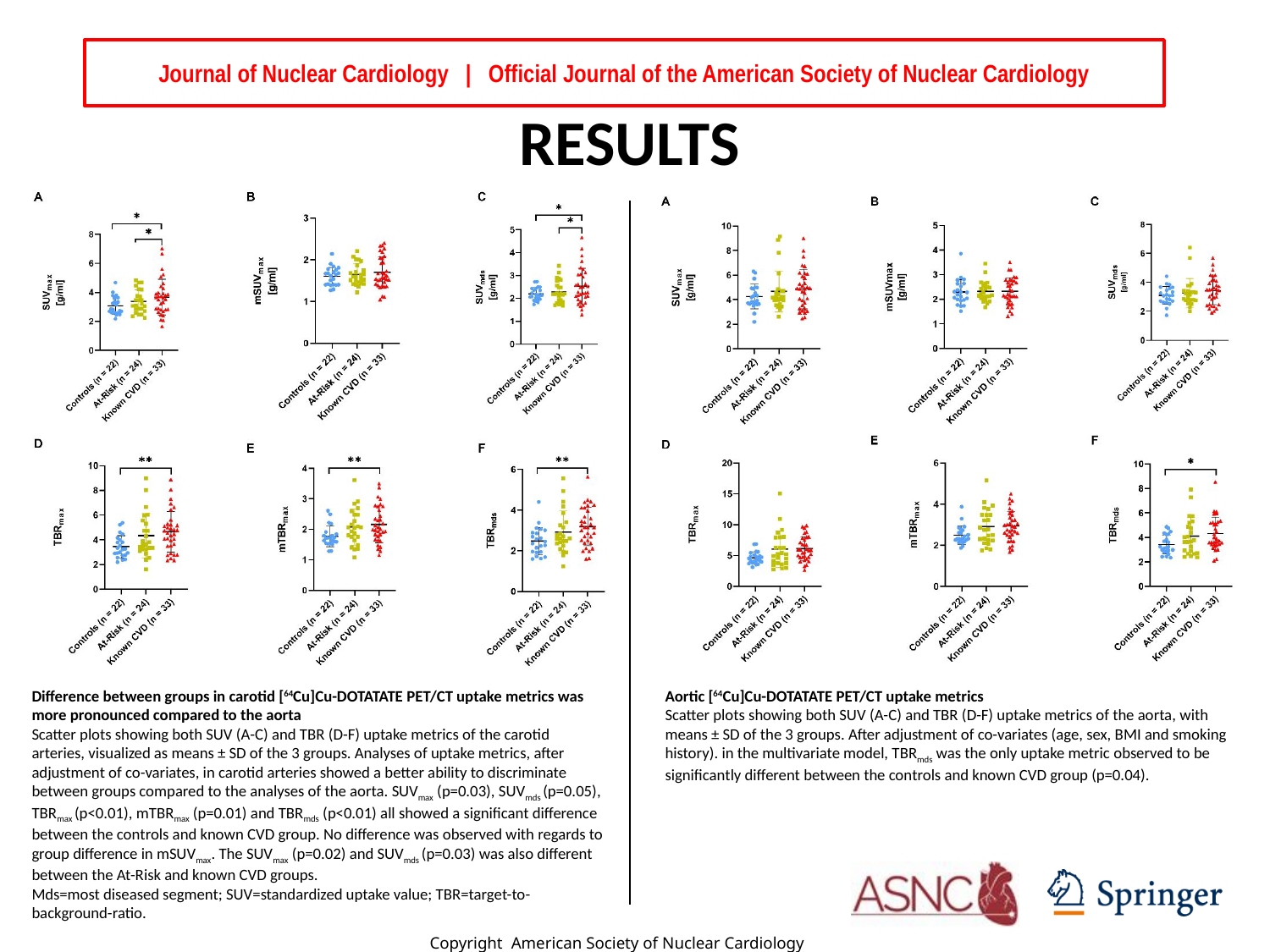

Journal of Nuclear Cardiology | Official Journal of the American Society of Nuclear Cardiology
# RESULTS
Difference between groups in carotid [64Cu]Cu-DOTATATE PET/CT uptake metrics was more pronounced compared to the aorta
Scatter plots showing both SUV (A-C) and TBR (D-F) uptake metrics of the carotid arteries, visualized as means ± SD of the 3 groups. Analyses of uptake metrics, after adjustment of co-variates, in carotid arteries showed a better ability to discriminate between groups compared to the analyses of the aorta. SUVmax (p=0.03), SUVmds (p=0.05), TBRmax (p<0.01), mTBRmax (p=0.01) and TBRmds (p<0.01) all showed a significant difference between the controls and known CVD group. No difference was observed with regards to group difference in mSUVmax. The SUVmax (p=0.02) and SUVmds (p=0.03) was also different between the At-Risk and known CVD groups.
Mds=most diseased segment; SUV=standardized uptake value; TBR=target-to-background-ratio.
Aortic [64Cu]Cu-DOTATATE PET/CT uptake metrics
Scatter plots showing both SUV (A-C) and TBR (D-F) uptake metrics of the aorta, with means ± SD of the 3 groups. After adjustment of co-variates (age, sex, BMI and smoking history). in the multivariate model, TBRmds was the only uptake metric observed to be significantly different between the controls and known CVD group (p=0.04).
Copyright American Society of Nuclear Cardiology

## Slide 5
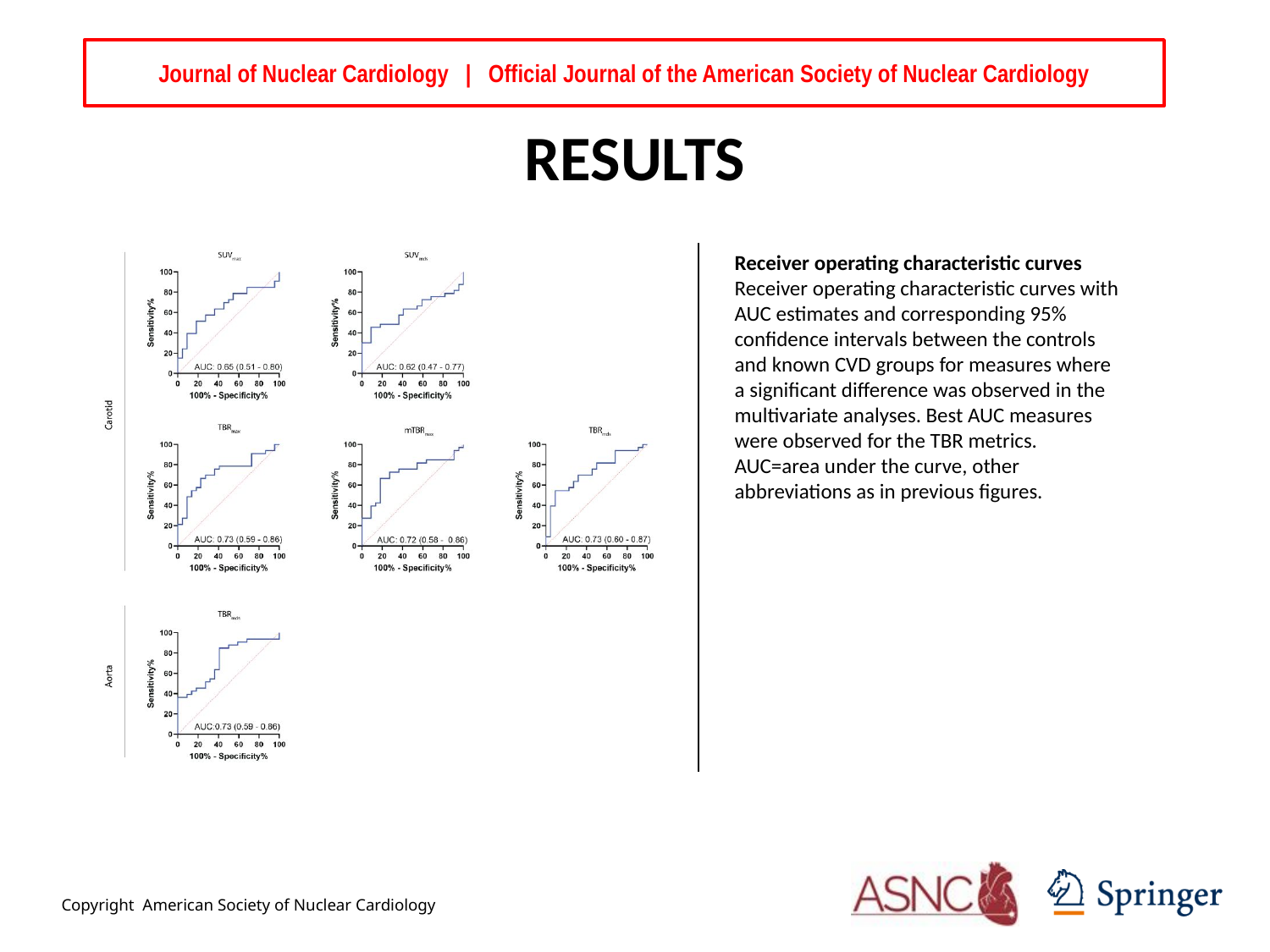

Journal of Nuclear Cardiology | Official Journal of the American Society of Nuclear Cardiology
# RESULTS
Receiver operating characteristic curves
Receiver operating characteristic curves with AUC estimates and corresponding 95% confidence intervals between the controls and known CVD groups for measures where a significant difference was observed in the multivariate analyses. Best AUC measures were observed for the TBR metrics.
AUC=area under the curve, other abbreviations as in previous figures.
Copyright American Society of Nuclear Cardiology

## Slide 6
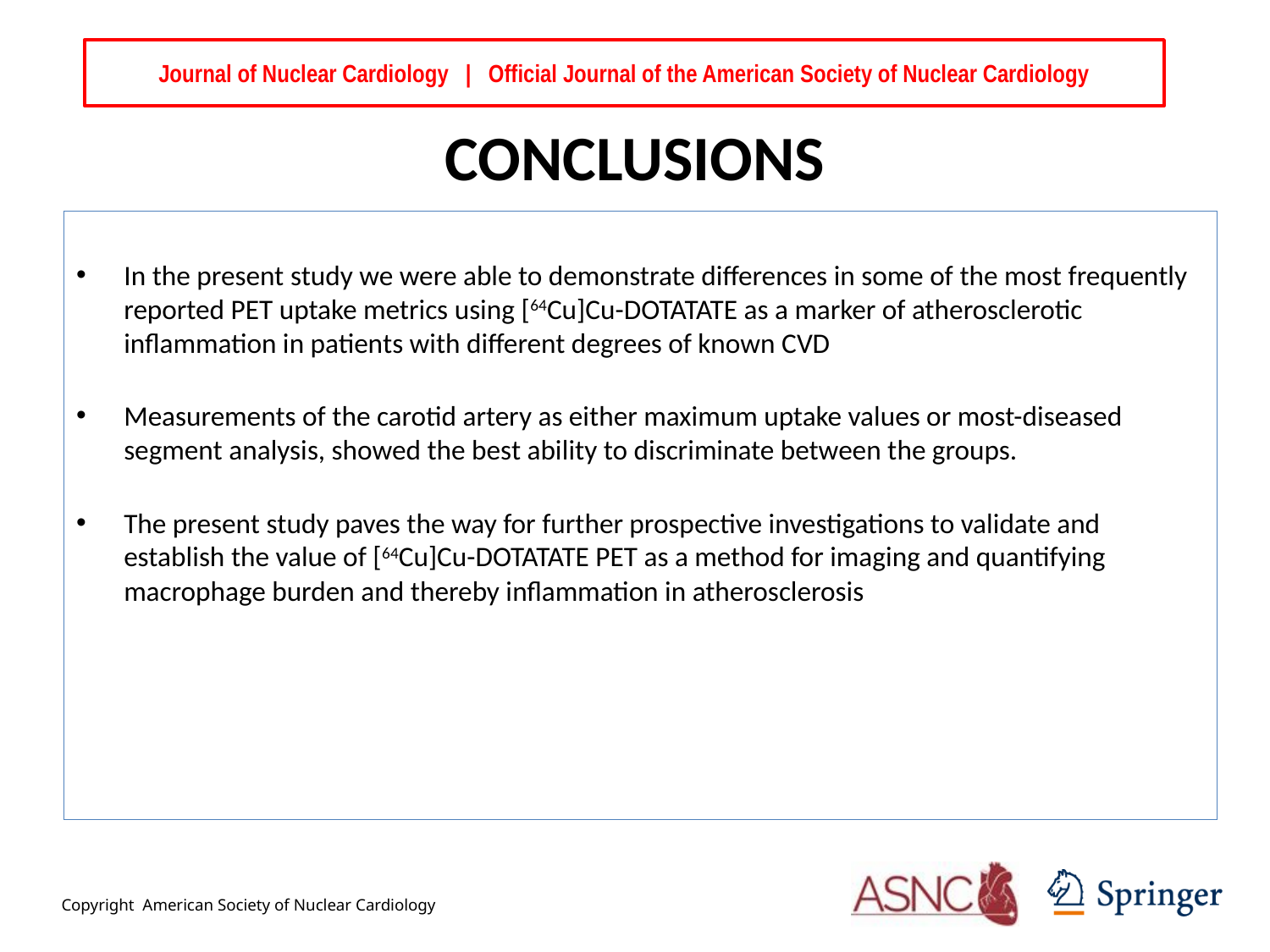

Journal of Nuclear Cardiology | Official Journal of the American Society of Nuclear Cardiology
# CONCLUSIONS
In the present study we were able to demonstrate differences in some of the most frequently reported PET uptake metrics using [64Cu]Cu-DOTATATE as a marker of atherosclerotic inflammation in patients with different degrees of known CVD
Measurements of the carotid artery as either maximum uptake values or most-diseased segment analysis, showed the best ability to discriminate between the groups.
The present study paves the way for further prospective investigations to validate and establish the value of [64Cu]Cu-DOTATATE PET as a method for imaging and quantifying macrophage burden and thereby inflammation in atherosclerosis
Copyright American Society of Nuclear Cardiology
